# Pathogenetic Action of High Potencies

**Published:** 1883-09

**Authors:** 

**Affiliations:** Alvensleben; Brooklyn


### ON PATHOGENETIC ACTION OF HIGH POTENCIES.

DR. BUCHMANN, ALVENSLEBEN.

*Translated by B. Fincke, M. D., from Allg. Hom. Zeitung, March* 6*th,* 1883. *Vol.* 106, *No.* 10, 12.

Every part of our body possessing the sense of taste is also capable of receiving the influence of medicines and of propagating their power upon all other parts.—*Hahnemann's Organon,* § *289.*

### I.

### PRELIMINARY REMARKS.

It is a very gratifying sign in the latest history of Homœopathy that there was such a keen interest in the action of high potencies at the last meeting of the Central Homœopathic Association in Stuttgart; for only the conviction of the efficacy of the highest potencies gives the necessary confidence in the efficacy of homœopathic doses in general. In regard to them it is very difficult for the younger physicians, as yet afflicted with traditional prejudices, to attribute any value, they being imponderables. Yet daily we have to combat powerful imponderable agencies often without success.

Several times already I have pointed out that the atomic theory of the physicist is by no means incompatible with our doctrine of high potencies. For further confirmation I quote some sentences from the late work of Gustav Hansemann, *The Atoms and their Motions*, which are apt to illustrate the physical process of homœopathic attenuation.

P. 65. "In all cases where the distance has exceeded the limit at which the action of the mutual protection comes to an end, the atoms can withdraw still more from each other in indefinable manner, hence the constituents of the presupposed simple system of simple material atoms lose all continuity, or, as we might call it, the system dissolves in the other."

P. 142. "The mutual protection of the material atoms from the shocks of the ether atoms ceases, and by supply of living force* explosion of the atoms ensues."

Pp. 74–75. "Every material atom at the surface of such a system can at any time by the action of special concussions with the neighboring material atoms receive such a great increase of living force that a separation from the system ensues. We will, therefore, divide the system of material atoms not into insoluble and soluble ones, but only into such as are more or less difficult to dissolve."

Dr. Katsch, whom I admire on account of his services to Homœopathy, has in Stuttgart accused the high potencies of mysticism, since they are prepared in an uncontrollable and mysterious manner. The veil has already been lifted by the article of colleague Dr. Schlegel, in the *Berliner Zeitschrift.*

Dr. Fincke, in Brooklyn, also has last year published the mode of preparation of his high potencies, which, during seventeen years in the United States, have been preferred on account of their surprising actions, and by this publication the doubt about their centesimality has been removed.

Since his process of fluxion seems to me eminently appropriate for the preparation of such high attenuations, I hope to do a service also to our German colleagues who are already interested in the nervo-analytical proving of Natr. mur.$^{4m}$ (Fincke) of Prof. G. Jaeger, by describing this process. (Here follows the translation from THE HOMŒOPATHIC PHYSICIAN, Vol. II, p. 217.)

Nobody will doubt that pure water used after the 6th potency is at least as good a vehicle for further attenuation as alcohol.

*By supply of living force the author understands also the assistance of motion, a force of which we also avail ourselves in our homœopathic triturations and succussions.

Dr. Katsch wants control of the preparation of high potencies, but this is impracticable even for the lower potencies. Just as we trust to the respectability of our homœopathic apothecaries, we also must put full confidence in those who in order to help suffering humanity have made it their tiresome, thankless task of life to make high potencies, as *Karsakoff*, *Jenichen*, and *Fincke* have done, and trust that each of them has endeavored in his own way to prepare "*lege artis*" efficacious remedies in that degree which he has given them on the label.

Dr. Katsch has had no success from his own high potencies prepared by himself and given in inflammations of the lungs and scarlatina. I admit readily that in certain acute diseases, especially if the application of a remedy which is generally recommended for them is concerned, lower attenuations may be more efficacious. But I deem the above-mentioned diseases, which without definite duration, also many times without medication, sooner or later get well, not fit for experimentation in order to pass judgment upon the efficaciousness of the high potencies; for these are not to be selected according to the categories of disease, and frequently special circumstances must turn the scale in their selection. If, however, *e. g.*, osseous ganglia of long standing, difficult hearing caused by chronic catarrh of the cavity of the tympanum, nasal polypi, several times operated upon unsuccessfully, a cataract, an intermittent with enlargement of the spleen and liver lasting over a year, and resisting medical applications etc.—if all these affections have been rapidly and permanently cured by one dose of one remedy in high potency, as I have observed, and partly published, then confidence in high potencies seems to me perfectly justified, and those who by principle abstain from their administration, as do the liberal eclectics of North America, who do not go beyond the 10th potency, miss their finest healing successes.

They do not trust to the hundreds of cures by high potencies which have been published in the journals, because they start from the premise that there are no more material atoms of the medicine in the 12th potency, therefore they conclude that the higher potencies must be inert, as Dr. C. Wesselhoefft has tried to make out in the *Rundschau.*

Several years ago I had Sanguinaria only in the 200th potency, and yet this became my main reliance against nervous headache, so that I fell not even into the temptation of using a later bought

AHZ 106 10,12

Zuchera fortis for attenuations, because the effect always promptly occurred a few minutes after taking.

That the highest potencies are able to act *pathogenetically*, even through the vial and the hand holding it, will be shown by the following experiments taken from the HOMŒOPATHIC PHYSICIAN, July, 1882, p. 298.

II.

(Here follow the provings of Lachesis by Dr. B. Fincke, *l. c.*)

III.

PROVING OF LACHESIS 5M (FINCKE) BY DR. BUCHMANN.

Dr. Fincke had been so kind as to give us, among other high potencies, a vial with globules of the five millionth centesimal potency of Lachesis, which he had made for curiosity's sake, since he does not *in praxi* give any preference to the millionth potency for the hundred thousandth. I showed the vial, January 14th, to my trusty prover, F. B., who had to suffer so much by the above mentioned proving of live quicksilver sealed up in a bottle and held in her hand. She saw with pleasure the small sugar-pellets, looking like fine granules of sand, and expressed her disbelief that such a high attenuation could have any effect whatever, though she had already experienced magically rapid healing action upon herself from high potencies. I asked her to keep the vial for a few minutes in her right hand, and she finally did so, though unwillingly, because she had not much time. To prevent any impatience on her part, I directed the conversation to other things of interest, when suddenly, about three minutes after taking the vial in her hand, her face was distorted, and she complained of spasmodic pain in the right hand, while at the same time the third and fourth fingers were drawn firmly around the vial. Heat running over the upper part of the body, with oppression around the margin of the lower ribs, and the desire to yawn without accomplishing it. The jaws are drawn asunder by force, the mouth is opened, she jumps up, anxiously tries to yawn, but cannot do it. At the same time water runs out of her mouth, tears fall over her cheeks, and she must hold the handkerchief before the nostrils in order to take up the fluid running from the nose. In short intervals when she complains of being tired, two more attacks of ineffectual yawning-spasms occur. This took about a quarter of an hour. I now put the vial in her left hand, and she yielded, saying that the attacks were probably over now. But she was mistaken. For after a few minutes suddenly cramp in the left hand came on, by which the hand was firmly closed. She jumps up with the cry: "I must choke," and offers me the left hand to take the vial out of it, because the spasm holds it fast. Then the same symptoms repeat themselves just the same as when she holds the vial in her right hand, lasting just as long and with the same violence.

The next day this prover, very much against her will, was induced to repeat the proving, and only on condition of putting the vial away as soon as it would affect her too much. It seemed to her now as if the vial caused some burning in the hand, but other symptoms which I expected with eagerness did not appear, in spite of holding the vial for a quarter of an hour. I regret that I did not then try a lower potency of Lachesis. When I took the vial from the hand of the prover it produced in my left hand for several minutes sensation of unusual coldness emanating from the vial.

Now, what will our German eclectics, to whom my proving of quicksilver, by induction, nineteen years ago appeared incredible, to whom already the Hahnemannian globules of the 30th potency as olfactory remedy are a monstrosity, say to *these* provings? One cannot imagine such yawning spasms where the water pours out of eyes, nose, and mouth, when they happen before your eyes. On the whole, it is very rare that the prover ever yawns; she was, besides, perfectly healthy; and it is necessary for hysterical attacks to be an habitual sufferer from hysteria, which she was not. The possibility of any simulation is entirely excluded. Every doubt of the action of the five millionth potency must vanish if we compare the symptoms which the indefatigable Dr. Fincke observed with the symptoms of the proving given above.

1. B. F., after taking on the tongue six globules Lachesis$^{71m}$ Fincke: spasm of the jaws, so that he could close his mouth only with difficulty, with spasmodic pains in the mandibular joints.

2. Mrs. T.—Spasmodic contraction of the third and fourth fingers of the right hand.

3. Miss C. F.—Repeated yawning, with fatigue.

4. Same.—Anxiety from the pit of the stomach.

No expert will raise the objection, that also at the proving on the second day symptoms of Lachesis should have appeared, since we have here to deal, not with a physical, but with a physiological experiment.

### I.

### Comments of Dr. B. Fincke.

The most valuable contribution to *Materia Medica Pura* by our transatlantic friend shows to the expert one important feature, the bearing of which on the benefit to mankind is indeed incalculable. When I read to Dr. P. P. Wells the passage that the second day no symptoms appeared, he at once said: " The susceptibility to Lachesis was exhausted." And so it certainly was in this case, at least for this potency. It is doubtful whether or not another potency would have brought out the same or other symptoms, but it does not alter the fact of the exhaustion of the susceptibility this time. This fact is available for the doctrine of prophylaxis, such as we practice when we give Belladonna for the prevention of scarlet fever, or vaccinate and re-vaccinate or give Vaccinin or Variolin in order to protect from future infection by small-pox. The principle is correct, and rests upon the very fact shown in this proving. The remedy given acts upon the organism in such a way that when any of those natural high potencies are brought to bear upon it by the prevailing epidemics, it finds the susceptibility for it exhausted, and cannot fasten and germinate and grow up in the epidemic group of diseases. Vaccination is nothing else but a crude proceeding upon the same principle, and since the prevention is offered to the system in the form of a great mass of palpable poison, the effect is often worse than the real small-pox disease would have been. Besides, the animal poison used does not exhaust the susceptibility of the system, because it is not strictly homœopathic, for it is not obtained from the similar natural vaccine-disease of the cow, but from the unnatural inoculation of matter derived from a cow many generations ago, through generations of calves which are not cows. There must be a reason why the vaccine-disease appears at the udder of the cow but not upon that of the heifer, nor on the mammary glands of male calves and oxen. Being, therefore, not homœopathic, it cannot have the required homœopathicity or the quality of prophylaxis by exhaustion of the susceptibility of the system for small-pox. It follows, that we must select that prophylactic which is the most similar to small-pox. This is undoubtedly the poison from the variolous pustule itself taken from a case which bears all the characteristics of this dreaded disease in perfection, provided it supervened upon an individual otherwise healthy when infected.

For a regular disease it is having its normal course, which repeats itself in every infected individual, only in a lighter or lower degree. No expert will raise the objection, that such a procedure belongs not to Homœopathy, reviving the old squabbles about isopathy, which should have found their end long ago. If I take a drop of lymph from a suitable small-pox patient at the height of the disease before suppuration takes place, and potentiate it not only to 30, but much higher, as surely a homœopathic remedy has been obtained as in the case of the innocent Silicea and of the vile poison of that snake Lachesis, which everybody can see in Morton's Museum in Philadelphia. And as much Hahnemann himself has said at the end of the first volume of his *Chronic Diseases.* If that argument of isopathy would hold good, we would not be allowed to use any remedies at all, for they are all more or less equal in action, and, if equal the better, but on opposite sides, and that makes them similar. Does not Lachesis produce lock-jaw and symptoms of it as we see even from the five millionth? And would you hesitate a moment to apply it in such a case where the very same, not merely similar, symptoms are present, and would you not expect a cure? Where is then the similarity? The symptoms on the healthy are equal to the symptoms on the sick, and that is the only simillimum which can be obtained, and it is to all purposes and ends equal. But there is no need of changing our similia similibus, because it is true and exactly states the difference between æqualia and similia, viz., that there is only this difference, that æqualia stands for the highest degree in the series of simility; they are the similima, than there can be nothing more similar without infringing upon the domain of identity, which would be a logical blunder. The equality as the sign of comparison does not concern us as homœopathicians, though it underlies the homœopathic law of cure. See *High Potencies and Homœopathics*, 1865, Tafel, pp. 37, 48, 49, 127.

Now the homœopathic high potency of the small-pox lymph is the proper prophylactic for variola. We must find out which is the proper potency and dose to give, and at what time. This prophylactic can be safely put in the hands of the million; it is always ready, always pure, and does not spoil, and it can never be exhausted. It may be given whenever there is fear of any infection, once every year or oftener, in single or repeated doses, whenever any fear of infection appears. It can only exhaust the susceptibility of the system for small-pox, and is a safe kind of variolation, destined to take the terrors out of this dreadful scourge if judiciously managed.

### II.

The following is the result of a neuro-analytical proving of Lachesis 5M (Fincke), with which the proving of Buchmann was made on the electro-magnetic method:

(*a*.) Ten observations upon myself in good health gave a sum of . . . . . . . . . . . 1363°

After taking about a dozen globules of Lachesis 5M (Fincke) on the tongue.

Ten observations the next half hour, commencing after one and a half minutes, gave . . . . . . . . 1477°
therefore an increase in the first half hour of 114°, giving a difference of +8.4 per cent.

Ten observations the next half hour . . . . . 1472°
again increase of 109°, or a difference of +8 per cent.

(*b*.) Ten observations upon a young lady, fifteen years, in good health, gave . . . . . . . . . 1700°

After half an hour from the beginning, took Lachesis 5M (Fincke), about a dozen globules on tongue. After one and a half minutes, ten observations for the next half hour gave 1866°
therefore an increase of 166°, or a difference of +9.2 per cent.

Ten observations the next half hour gave . . . 1862°
hence an increase of 162°, or a difference of +9 per cent.

The provings were made two and two and a half hours after dinner—the first from 3½ to 5½ P. M., the second from 3 to 5 P. M.

### III.

Dr. Katsch is a representative of that class of physicians who abstain from the use of high potencies under the pretense of not knowing whether they really are what their labels indicate, and who cry for a control of the preparation of high potencies, which cannot be had, in order to be excused from being hampered any more with that unpleasant and difficult subject. What they are driving at in truth is very clear. They want to be relieved from the responsibility regarding the value of their preparations, and try to shift it upon the shoulders of the apothecary, the respectability of whom cannot be doubted. But, alas! look at the transactions of the American Institute for 1882, and the Dr. Katsches must be very much disappointed when they read there in a portly volume, with most improved metallic corners, in excellent print, what the then President had to say upon that topic.

Dr. Edward Smith has succeeded in assaying the different triturations of Aurum metallicum up to the 30th decimal, and with the most startling results. When Professor Smith sent me a "button" of pure gold obtained from assaying the 30th trituration of Aurum, large enough to handle and examine, which resisted boiling in nitric acid, and when I remembered how diligently our colleagues, Dr. Wesselhoefft and others, had been for years searching for this valuable article with the assistance of the microscope, and that the decimal trituration of Aurum properly prepared should not contain gold at all visible, I believed there must have been some mistake in the labeling of this particular preparation. It was then suggested that, as President of the Institute, I should furnish the trituration for examination. Ordering from nine reputable homœopathic pharmacies preparations of the 1, 2, 3, 4, 5, 6, and 30 of Aurum, I carefully removed all labels and evidences of their origin, marked the corks by letters and numbers, carefully registering each in my book, and forwarded them to Professor Smith. The results of these examinations will be given to the Institute at the proper time, and prove conclusively that triturations of gold as sold above the 7 decimal are totally unreliable, the 30th and even the 60th yielding the same amount of gold as was found in the 7th.

Owing to this testimony, corroborated by the details of the examinations furnished by Dr. Smith, and still more aggravated by facts coming out at the discussion (p. 668), the confidence in those nine reputable homœopathic pharmacies must be considerably shaken. But their names are lovingly concealed, and the physician remains in the dark and does not know to whom to apply in order to escape such impostures as are here practiced in the open day. The ostensible reason for these extensive examinations is, "not to expose the name of any of the pharmacists, but to stimulate them all to put upon the market better drugs, and as they have been holding the homœopathic profession in the hollow of their hand for a number of years, they may now be willing to drop it, and allow us to indicate the course and establish the rule." Here is a position for the homœopathic physicians and members of the Institute, assigned by the gentleman at the time in the chair. Every homœopathician must decline such an ignoble position. The examination of the products of the reputable pharmacies may be important for the brethren who are content with the first three decimal triturations, which, we are informed, are generally found to be correctly labeled, but they are of no earthly use to science, and it shows a deplorable status of the great majority of the homœopathic body on this continent if such efforts are made merely "to hold the pharmacists in position."

Here is an example for the Dr. Katsches to consider. If the pharmacists are not even to be trusted for their decimal triturations, which can be examined by assaying them and by putting them under the microscope, how can you trust them if it comes to high potencies for which there is no assaying and no microscopic test possible, the efficaciousness of which can only be found by trying them upon the healthy and sick and by the latest test of Neural Analysis? But, alas, what has been found out in the lower region of decimal triturations has likewise occurred in the high region of centesimal potencies. For as soon as the fluxion process was known, certain parties who after the example of the courtesies extended in the lower region may be left unnamed, have taken advantage of it, and prepared medicines of which, in regard to potency, number, and scale, nobody knows what they are, and which certainly do not come up to the fabulous heights assigned to them. It seems, then, we on the upper side of the question are no better off than those on the lower.

In this dilemma there are only two possibilities of getting out of it. Either we trust to somebody who deserves it after having faithfully tried what he has produced, or dear, Dr. Katsch, you must follow the way that Hahnemann has traveled before and which with many others you also have already trodden, viz.: you must make your own remedies. Then there is no question of confidence in what you have done yourself. If, then, you have deceived yourself, and the potencies don't turn out as potent as others, you cannot find fault with anybody else. But the idea of creating a control for the preparation of high potencies by dictation of institutes and societies militates against the free spirit of science, and cannot be countenanced for a single moment. The control must be in the integrity of those who have a scientific and practical interest in this matter, and in the necessary consequences which science generally draws immediately from new discoveries.

The inferences drawn by the President of the Institute for 1882 from the misdemeanors of the nine pharmacists are disingenuous in a high degree, because he merges Hahnemann's high conception of homœopathics into the low prejudiced reasoning of his own mind.

It is not true "that the absence of a uniform standard in the preparation of drug attenuations has afforded the opportunity for the surreptitious introduction of methods that were never dreamed of by Hahnemann and that should have no place in scientific medicine." It is true that Hahnemann was no dreamer, but to the end of his long life he tried to make his potencies higher and approved of those then in existence. The uniform standard was laid down by him unmistakably in the centesimal scale, so this cannot be absent. But the enemies of Hahnemann, who always quote him for their falsehoods, tried to introduce the decimal scale, long disowned by its inventor, and that is the uniform standard they are aiming at. Reading it in this way, the absence of the centesimal legitimate standard has not given rise to surreptitious introduction of new methods, but it was only, in accordance with the Hahnemannian uniform standard, used for those methods by which alone higher potencies than known before could be obtained. The accusation that these methods have been surreptitiously introduced depends again upon a falsehood which is in the same vein with the sneer of bottle washing, and can only be laid to that exuberance of fanaticism with which medical history abounds. The truth is, the methods alluded to have been openly and carefully introduced as the legitimate offspring of scientific investigation. This investigation has been going on for many years, but the President is entirely innocent of it, for timidly he asks: "Shall we not then investigate for ourselves rather than allow others to attend to that matter for us?" Just as in the matter of pharmaceutics he gives up his right to the apothecary, so now the fear that "modern allopathy will enter the arena against Homœopathy" and look "after our provings, pharmacological processes, and also our preparation," drives him again in the arms of the pharmacies, for "our pharmacists cannot escape much longer and the American Institute will have to make a record on the subject." What he actually means is, however, not a candid elucidation of the subject, but a decree of the Institute, a Pope's bull, to restrict the homœopathic practitioners to the tenth centesimal in their practice, because ninety-nine out of every hundred rely upon triturations and dilutions within the range ending at the tenth decimal.

Enough! "*Quousque tandem abutere patientia nostra.*" A homœopathic physician, even when in the chair of the Institute, who calls infinite dilution an absurd doctrine never taught by Hahnemann, and who says that Hahnemann understood the subject in the sense of the President when he advised a limit to drug attenuations, deserves no more respect than the devil quoting Scripture. For Hahnemann not only advised physicians to use the 30th centesimal, which is already infinitesimal (mark, not the 10th and 12th of Wesselhoefft-Breyfogle), only for the purpose of making observations, but he also admonished us in his writings to make higher potencies when the case should require it, and to his last days he tried to carry his potencies higher and applied them in practice, and besides approved of others then in existence. And it is a fact which cannot be repeated often enough, that in all his multifarious writings he inculcates the necessity of infinite dilution for the remedies well-selected according to homœopathic law.

To such perversions of truth, therefore, it is not necessary to add a simple protest, because they carry the germ of their refutation in themselves.

V.

It would be interesting to know whether any of those nine participated in the famous Milwaukee test, and it would on the whole, be well to have their names.

VI.

Curious it is to see how the champions of the low decimal triturations carry on, trying to bully the homœopathicians out of their impregnable position by attempting to dictate the uniform standard of a decimal scale and a limit to the 10th or 12th centesimal potency at a time when Neural-Analysis, the highest acquirement that homœopathics could hope ever to obtain, shows the action of the highest potencies through physical means. "*L' extremes se touchent.*"

VII.

But in the same breath, when high potencies are urged upon these pseudo-homœopaths, they very liberally claim the whole scale for their use, though in point of fact they rise rarely higher than the first decimals. He may find consistency in this behavior who can! But we accept this allowance cheerfully, adding to it, however, that we find it founded on the very principle of *similia similibus*, that only medicines should be given which have no body to them except the inert vehicle which keeps them, viz.: potencies.

From the facts stated above, it appears that the series of potencies which in Hahnemann's time extended no higher than the 30th centesimal gradually has risen higher and higher till it has reached the five millionth centesimal, a scale which must satisfy the most fastidious of those homœopathic practitioners who maintain that the whole scale of a remedy, from the crude substance up to the highest potency, must be at the disposition of the physician, as is also taught at least in one homœopathic college. It is now to be expected that those scientists will also point out the indication for the potency to be selected in the given case, ranging now between the crude substance and the five-millionth centesimals, since the hackneyed rule to give low potencies in acute and high potencies in chronic diseases does not hold good any more.

For in the proving above given the 5M produced symptoms of great acuteness, and it stands to reason that when it is able to produce acute symptoms it must also be able to cure them. If the prover had been afflicted with the symptoms given the 5M of Lachesis would no doubt have been the right potency to cure her. For *similia similibus curantur.*

VIII.

*Ceterum censeo macrodosiam esse delendam.*

BROOKLYN, *April* 17th, 1883.

